# Itaconate Alters Succinate and Coenzyme A Metabolism via Inhibition of Mitochondrial Complex II and Methylmalonyl-CoA Mutase

**DOI:** 10.3390/metabo11020117

**Published:** 2021-02-18

**Authors:** Thekla Cordes, Christian M. Metallo

**Affiliations:** Department of Bioengineering, University of California, 9500 Gilman Drive, La Jolla, San Diego, CA 92093, USA; tcordes@ucsd.edu

**Keywords:** itaconate, succinate, methylmalonate, succinate dehydrogenase, isotopic tracing, TCA cycle metabolism, odd-chain fatty acids (OCFAs), branched-chain amino acids (BCAA), acetyl-CoA, propionyl-CoA, itaconyl-CoA, vitamin B_12_

## Abstract

Itaconate is a small molecule metabolite that is endogenously produced by cis-aconitate decarboxylase-1 (ACOD1) in mammalian cells and influences numerous cellular processes. The metabolic consequences of itaconate in cells are diverse and contribute to its regulatory function. Here, we have applied isotope tracing and mass spectrometry approaches to explore how itaconate impacts various metabolic pathways in cultured cells. Itaconate is a competitive and reversible inhibitor of Complex II/succinate dehydrogenase (SDH) that alters tricarboxylic acid (TCA) cycle metabolism leading to succinate accumulation. Upon activation with coenzyme A (CoA), itaconyl-CoA inhibits adenosylcobalamin-mediated methylmalonyl-CoA (MUT) activity and, thus, indirectly impacts branched-chain amino acid (BCAA) metabolism and fatty acid diversity. Itaconate, therefore, alters the balance of CoA species in mitochondria through its impacts on TCA, amino acid, vitamin B_12_, and CoA metabolism. Our results highlight the diverse metabolic pathways regulated by itaconate and provide a roadmap to link these metabolites to potential downstream biological functions.

## 1. Introduction

Metabolism coordinates the conversion of available nutrients toward energy, biosynthetic intermediates, and signaling molecules to mediate biological functions. Dysregulation of metabolic pathways, via environmental stress, genetic perturbation, or inflammatory signaling, contributes to diverse metabolic diseases. Cells reprogram metabolism to control the synthesis of metabolites to modulate biochemical processes and cell function. However, accumulation or treatment of a single molecule or metabolite can influence diverse pathways throughout the biochemical network, making it challenging to mechanistically define how small molecules control cell function. 

Itaconate is a dicarboxylic acid synthesized de novo under pro-inflammatory conditions to combat microbial infections [1,2]. The molecule has emerged as a key regulator influencing microbial metabolism and endogenous pathways to control immune cell function [3,4,5,6,7,8]. Specifically, itaconate inhibits succinate dehydrogenase (SDH) activity, resulting in intracellular succinate accumulation [9,10,11]. Further, itaconate is metabolized to itaconyl-Coenzyme A (CoA) that inactivates mitochondrial coenzyme B_12_ (adenosyl-cobalamin), resulting in decreased methylmalonyl-CoA mutase (MUT) activity [12,13]. 

Given the manifold impacts of itaconate on central carbon metabolism and immune cell function, itaconate and its derivatives could have therapeutic potential that warrants further exploration. For example, itaconate is an anti-inflammatory [10,14,15] molecule and has antimicrobial [1,2,16,17] as well as antiviral [18,19,20] properties. Treatments with itaconate derivatives buffer disease severity in models of sepsis [14], Zika virus infection [18], psoriasis [15], and fungal keratitis [21]. Further, we and others have observed that exogenous itaconate and dimethylitaconate (DMI) can mitigate reperfusion injuries in vivo [10,22]. By exploiting the SDH inhibitory function of itaconate to limit damaging succinate accumulation [23,24], itaconate treatment allows mitochondria to gradually “awaken” and prevent cellular injury [22,25]. Itaconate is also an endogenous metabolite accumulating in mM levels inside a cell under pro-inflammatory conditions [1,7,26], and plasma itaconate concentrations correlate with disease activity in patients of with rheumatoid arthritis [27]. A focused analysis on the metabolic consequences of cells treated with itaconate is, therefore, warranted.

Here, we applied stable isotope tracing and mass spectrometry to quantify the metabolic impacts of itaconate in various cell types. We observed that itaconate is a competitive and reversible SDH inhibitor influencing succinate metabolism. In addition, we observed that itaconate is metabolized into itaconyl-CoA, which modulates MUT-dependent branched-chain amino acid (BCAA) metabolism. As a result, itaconate also alters the balance of CoAs, which also impacts fatty acid diversity. Collectively, we identified itaconate as a metabolic regulator influencing diverse biochemical pathways that may further impact cell biology and function in health and disease. 

## 2. Results

**Figure 1:**
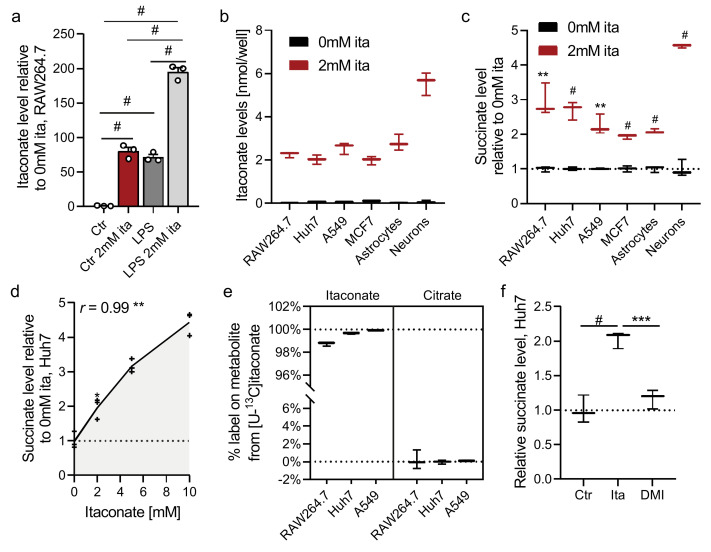
Itaconate promotes succinate accumulation in diverse cell types. (**a**) Itaconate levels in RAW264.7 cells exposed to LPS or 2 mM exogenous itaconate (ita) for 24 h compared to control condition (Ctr). (**b**,**c**) Itaconate (**b**) and succinate (**c**) levels in cells exposed to 2 mM itaconate for 48 h. (**d**) Pearson correlation coefficient (*r*) of itaconate and succinate levels in Huh7 cells exposed to increasing itaconate concentrations for 48 h. (**e**) Labeling on itaconate and succinate in cell lines cultured in the presence of 2 mM [U-^13^C_5_]itaconate. (**f**) Succinate levels in Huh7 cells exposed to 2 mM itaconate or 62.5 µM dimethyl-itaconate (DMI) for 48 h. Data are depicted as box and whiskers (**b**,**c**,**e**,**f**) or mean (**d**) ± s.e.m. (**a**) obtained from 3 cellular replicates. Students *t*-test (**c**) or one-way ANOVA (**a**,**f**) with no adjustment for multiple comparisons. * *p* < 0.05, ** *p* < 0.01, *** *p* < 0.001, # *p* < 0.0001.

To better understand how itaconate modulates mammalian metabolism, we cultured diverse cell types in growth media containing exogenous itaconate and quantified changes in the metabolome using mass spectrometry and tracing approaches. Notably, we observed that endogenous itaconate levels in LPS-activated RAW264.7 macrophages are similar to levels achieved in non-activated cells cultured in the presence of 2 mM exogenous itaconate (Figure 1a). This result indicates that cells take up extracellular itaconate and such treatments cause near-physiological accumulation in cells (albeit in potentially different cellular compartments). We previously observed that 2 mM itaconate could be readily achieved in plasma via infusion [22], further supporting the physiological relevance of this concentration.

### 2.1. Itaconate Promotes Succinate Accumulation in Diverse Cell Types

We next cultured several mammalian cell types in the presence of 2 mM exogenous itaconate, including RAW264.7 immune cells, hepatocarcinoma cells (Huh7), lung adenocarcinoma (A549), breast cancer (MCF7), and primary brain cells (i.e., astrocytes and neurons). While itaconate was not detectable in non-treated cells, we observed significant accumulation in treated cells (Figure 1b and Figure S1a). We also observed that the addition of unlabeled itaconate diluted labeling of endogenously synthesized ^13^C-itaconate in LPS-activated RAW264.7 macrophages cultured with [U-^13^C_6_]glucose or [U-^13^C_5_]glutamine (Figure S1b). These data further indicate that exogenous itaconate is transported into the cells tested.

As expected, itaconate significantly increased intracellular succinate levels (Figure 1c and Figure S1c), and succinate correlated positively with itaconate levels (Figure 1d) indicating impaired SDH activity. Since itaconate degradation has been observed in isolated mitochondria from liver [28,29], we next analyzed if cells use itaconate as a carbon source to fuel tricarboxylic acid (TCA) cycle metabolism and succinate synthesis. We, therefore, cultured cells with [U-^13^C_5_]itaconate and quantified enrichment on TCA intermediates. While itaconate was entirely labeled, we did not detect appreciable isotope enrichment on succinate or other TCA cycle intermediates (Figure 1e, Figure S1d,e). Thus, itaconate caused succinate accumulation in cells of various tissue origin but was not metabolized further to fuel carbons into the TCA cycle under these conditions. DMI did not influence succinate or intracellular itaconate levels (Figure 1f and Figure S1f). Further, DMI was cytotoxic at concentrations above 100 µM (Figure S1g), while itaconate had no impact on cell viability (even at concentrations above 10 mM) (Figure S1h). These data further highlight that a key consequence of itaconate accumulation is an increase in succinate levels, which contrasts that observed with DMI.

### 2.2. Itaconate Is a Competitive and Reversible SDH Inhibitor in Diverse Cell Types

**Figure 2:**
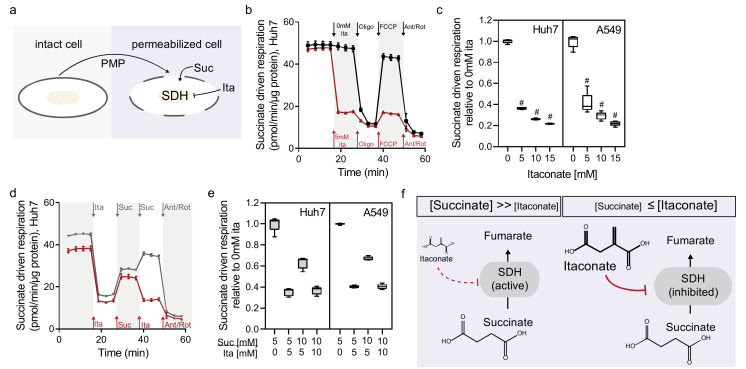
Itaconate is a competitive and reversible SDH inhibitor in diverse cell types. (**a**) Schematic depicting plasma membrane permeabilizer reagent (PMP) to access mitochondrial respiration in permeabilized cells. (**b**) Succinate-driven respiration in Huh7 cells in the presence (red) or absence (black) of itaconate (ita). (**c**) Succinate-driven respiration with increasing itaconate concentrations in Huh7 and A549 cells. Significance is depicted compared to 0 mM itaconate. (**d**) Succinate-driven respiration after serial addition of Ita/Suc/Ita (red) or Ita/Suc/Suc (grey) in HuH7 cells. (**e**) Succinate driven respiration after serial addition of itaconate or succinate in Huh7 and A549 cells. (**f**) Schematic depicting the competitive inhibition of itaconate on succinate dehydrogenase (SDH) activity. Data depict mean ± s.e.m. (**b**,**d**) or box and whiskers (**c**,**e**) obtained from 5 cellular replicates. One-way ANOVA (c) relative to 0 mM itaconate with no adjustment for multiple comparisons and # *p* < 0.0001.

SDH is a critical enzyme involved in TCA cycle metabolism and electron transport chain (Complex II) and impaired enzyme activity leads to succinate accumulation [9,10,11,22]. Therefore, we quantified the impact of itaconate on oxygen consumption in real-time with a Seahorse analyzer using Huh7 and A549 cells. To directly access mitochondrial Complex II activity, we permeabilized cells using perfringolysin O and offered cells succinate and itaconate at various concentrations (Figure 2a). The addition of itaconate rapidly decreased succinate-driven oxygen consumption in Huh7 and A549 cells (Figure 2b and Figure S2a), indicating that itaconate inhibited SDH activity instantly. We also observed that the sequential addition of itaconate decreased respiration in a dose-dependent manner. In comparison, sequential addition of succinate did not further increase respiration (Figure 2c and Figure S2b,d). These data indicate that SDH activity is not limited by substrate availability and itaconate influences this activity in a dose-dependent manner. 

The chemical structures of itaconate and succinate are similar, suggesting the two compounds compete for access to the SDH binding site. Notably, the Michaelis constant (K_M_) for succinate is 0.29 mM while the Ki for itaconate inhibition is 0.22 mM [10]. Therefore, we hypothesized that SDH inhibition by itaconate is highly dynamic and reversible if cells were offered excess succinate. We observed that itaconate-induced SDH inhibition was reversible after the addition of succinate in Huh7 cells indicating that SDH activity is saturated (Figure 2d and Figure S2e). We also validated our findings in A549 cells (Figure 2e and Figure S2d), indicating that itaconate is a competitive and reversible inhibitor influencing SDH activity in diverse cell types. Further, the rapid SDH inhibition is dependent on succinate availability suggesting a highly dynamic and reversible regulation of SDH activity by itaconate (Figure 2f).

### 2.3. Itaconate Influences Glutamine Metabolism

**Figure 3:**
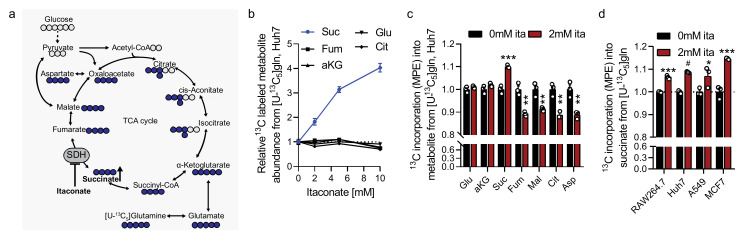
Itaconate influences glutamine metabolism. (**a**) Schematic depicting carbon incorporation into tricarboxylic acid (TCA) cycle intermediates from [U-^13^C_5_]glutamine (blue). Open circles depict ^12^C, closed circles ^13^C carbon. (**b**) Labeled metabolite level (depicted as abundance times mole percent enrichment (MPE)) from [U-^13^C_5_]glutamine in Huh7 cells cultured in the presence of 2 mM itaconate for 4 h. (**c**) ^13^C incorporation (mole percent enrichment, MPE) into metabolites from [U-^13^C_5_]glutamine in Huh7 cells cultured for 48 h. (**d**) ^13^C incorporation into succinate from [U-^13^C_5_]glutamine in diverse cell types in the presence of 0 mM or 2 mM itaconate after 48 h. Data are depicted as mean ± s.e.m. obtained from 3 cellular replicates. Students *t*-test with no adjustment for multiple comparisons and * *p* < 0.05, ** *p* < 0.01, *** *p* < 0.001, # *p* < 0.0001.

To gain more in-depth insights into mitochondrial metabolic compensation induced by itaconate, we cultured cells in the presence of [U-^13^C_6_]glucose or [U-^13^C_5_]glutamine and quantified labeling on TCA cycle intermediates (Figure 3a and Figure S3a). We observed that after a short culture of 4 h, itaconate significantly increased glutamine-derived carbons into succinate while other TCA cycle intermediates were not affected (Figure 3b). We also observed decreased carbon incorporation into TCA cycle intermediates downstream of SDH, specifically fumarate, malate, aspartate, and citrate after culture in [U-^13^C_5_]glutamine tracer medium for 48 h (Figure 3c and Figure S3b–d). Further, cells cultured in the presence of [U-^13^C_6_]glucose had increased ^13^C incorporation into metabolites downstream of SDH (that is, fumarate, malate, citrate, and aspartate) (Figure S3e–h). Indeed, the predominant change in citrate was increased M+5 isotopologue (Figure S3f), which are indicative of pyruvate carboxylase activity that would be expected to increase in response to decreased SDH activity [30,31]. Collectively, our data indicate that itaconate drives cells to accumulate glutamine-derived succinate in diverse cell types (Figure 3d).

### 2.4. Itaconate Alters Nitrogen and Branched-Chain Amino Acid Metabolism

**Figure 4:**
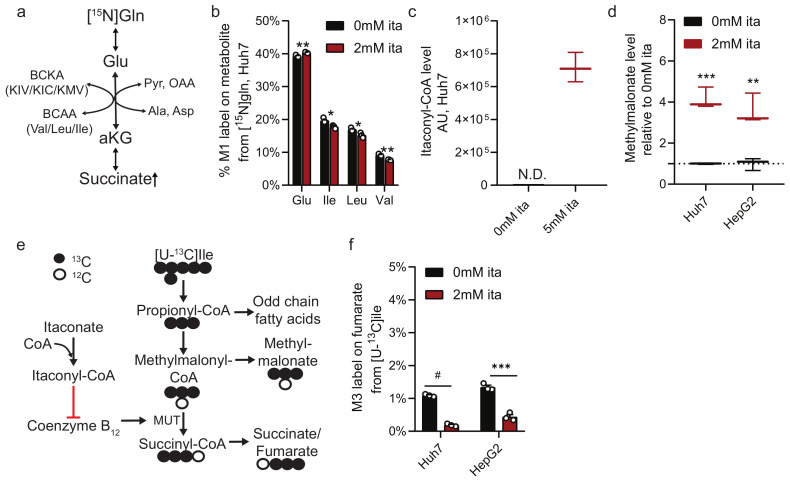
Itaconate influences methylmalonyl-coenzyme A (CoA) mutase (MUT) dependent BCAA metabolism. (**a**) Schematic depicting nitrogen exchange from [α-^15^N]glutamate on branched-chain amino acids (BCAA) and branched-chain keto acids (BCKA). (**b**) Labeling on amino acids from [α-^15^N]glutamine in Huh7 cells cultured for 48 h in the presence of 2 mM itaconate. (**c**) Itaconyl-CoA levels depicted as ion counts (m/z = 880.1379) in Huh7 cells. N.D. not detectable. (**d**) Methylmalonate levels in Huh7 or HepG2 cells exposed to 2 mM itaconate for 48 h compared to 0 mM itaconate (dotted line). (**e**) Schematic depicting methylmalonyl-CoA mutase (MUT) dependent BCAA catabolism. Open circles depict ^12^C, closed circles ^13^C carbons from [U-^13^C_6_]isoleucine tracer. (**f**) M3 label on fumarate from [U-^13^C_6_]isoleucine in Huh7 and HepG2 cells exposed to 2 mM itaconate for 48 h. Data are depicted as box and whiskers (**c**,**d**) or mean ± s.e.m. (**b**,**f**) obtained from 3 cellular replicates. Students *t*-test with no adjustment for multiple comparisons and * *p* < 0.05, ** *p* < 0.01, *** *p* < 0.001, # *p* < 0.0001.

Given that itaconate influenced glutamine metabolism, we next quantified the impact of itaconate on amino acid and nitrogen metabolism. Huh7 cells were cultured in the presence of [α -^15^N]glutamine to quantify abundances and labeling on amino acids (Figure 4a). In contrast to nonessential amino acids that showed increased labeling in itaconate-treated cultures (Figure S4a), isotope enrichment of the branched-chain amino acids (BCAA) leucine, isoleucine, and valine were decreased (Figure 4b). We recapitulated this phenotype in HepG2 cells (Figure S4b), indicating that itaconate altered the exchange flux catalyzed by BCAA transaminase (BCAT) that may further influence BCAA metabolism.

Itaconate is also metabolized to itaconyl-CoA and interacts with coenzyme B_12_ to inhibit B_12_-dependent methylmalonyl-CoA mutase (MUT) activity [12,13], a pathway fueled by BCAAs [32]. Indeed, upon treatment with itaconate, Huh7 cells showed a peak with m/z of 880.1379, which suggests accumulation of itaconyl-CoA (Figure 4c). Furthermore, we observed significantly increased methylmalonate levels upon addition of itaconate (Figure 4d), even in the presence of additional Vitamin B_12_ (Figure S4c). These data support a role for itaconate in modulating propionate metabolism through its influence on Coenzyme B_12_ and MUT activity. 

Since MUT catalyzes the formation of succinyl-CoA from sources of propionyl-CoA such as valine or isoleucine, we hypothesized that itaconate influences MUT-dependent anaplerosis. We next traced Huh7 and HepG2 cells with [U-^13^C_6_]isoleucine and quantified M3 label on fumarate to examine BCAA catabolic pathway contribution to the TCA cycle (Figure 4e). Although labeling was low, itaconate significantly decreased fumarate enrichment from ^13^C-labeled isoleucine (Figure 4f), even in the presence of additional Vitamin B_12_ (Figure S4d). These data support a role for itaconate in modulating a critical node of metabolism linking BCAA catabolism, coenzyme B_12_, and TCA anaplerosis. 

### 2.5. Itaconate Alters CoA Metabolism and Fatty Acid Diversity

**Figure 5:**
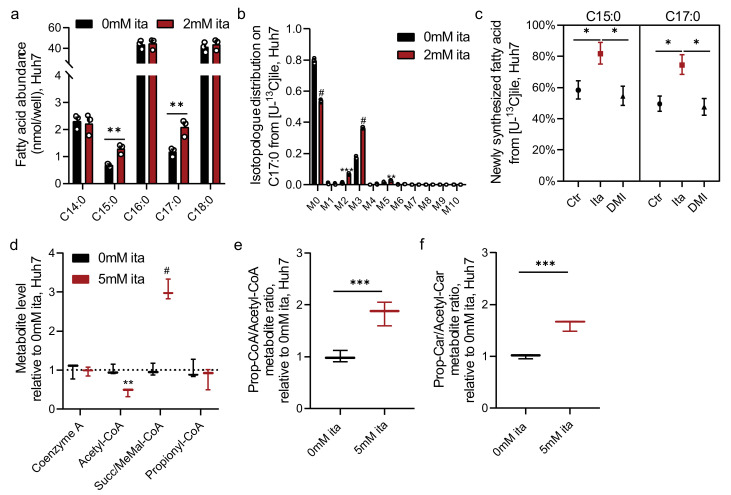
Itaconate alters fatty acid diversity and CoA metabolism. (**a**) Levels of fatty acids in Huh7 cells cultured for 48 h in the presence of 2 mM ita. (**b**) Isotopologue distribution on C17:0 from [U-^13^C_6_]isoleucine in Huh7 cells after 48 h. (**c**) Newly synthesized C15:0 and C17:0 from [U-^13^C_6_]isoleucine in Huh7 cells exposed to itaconate or dimethyl-itaconate (DMI) for 48 h. (**d**) Levels of CoA species in Huh7 cells exposed to 5 mM itaconate for 48 h compared to 0 mM itaconate (dotted line). MeMal-CoA; Methylmalonyl-CoA. (**e**) Propionyl-CoA to acetyl-CoA metabolite ratio in Huh7 cells after 48 h. (**f**) Propionyl-carnitine to acetyl-carnitine metabolite ratio in Huh7 cells after 48 h. Data are depicted as box and whiskers (**d**–**f**), mean ± s.e.m. (**a,b**) or 95% confidence intervals from ISA model (**c**) obtained from 3 cellular replicates. Students *t*-test (**a**,**b**,**d**–**f**) with no adjustment for multiple comparisons * *p* < 0.05, ** *p* < 0.01, *** *p* < 0.001, # *p* < 0.0001. Significance was considered as non-overlapping confidence intervals for (**c**).

Our data indicate that itaconate modulates MUT-dependent BCAA catabolism downstream of propionyl-CoA (Figure 4). We, and others, previously demonstrated that alterations in this pathway influence the synthesis of odd- and monomethyl branched-chain fatty acids (OCFAs, mmBCFAs) [32,33,34]. To determine if itaconate also impacts propionyl-CoA metabolism and OCFA levels, we quantified total fatty acid abundances in Huh7 cells. Notably, itaconate specifically increased abundances of C15:0 and C17:0 OCFAs while ECFAs (C14:0, C16:0, and C18:0) were not impacted (Figure 5a). Next, we employed Isotopomer Spectral Analysis (ISA) to quantify the contribution of ^13^C labeled precursor into the lipogenic CoA pool as well as newly synthesized fatty acids (Figure S5a) [32,35]. We cultured cells in growth medium containing [U-^13^C_6_]isoleucine that labels odd-chain fatty acids via propionyl-CoA pathway (Figure 4e and Figure S5a). The addition of itaconate significantly increased labeling on OCFA C17:0 (Figure 5b) and fractional synthesis of OCFAs (C15:0 and C17:0) (Figure 5c), while the relative contribution to each CoA pool was not impacted (Figure S5b). Notably, DMI treatment did not impact methylmalonate levels (Figure S5c) or OCFA synthesis (Figure 5c).

**Figure 6:**
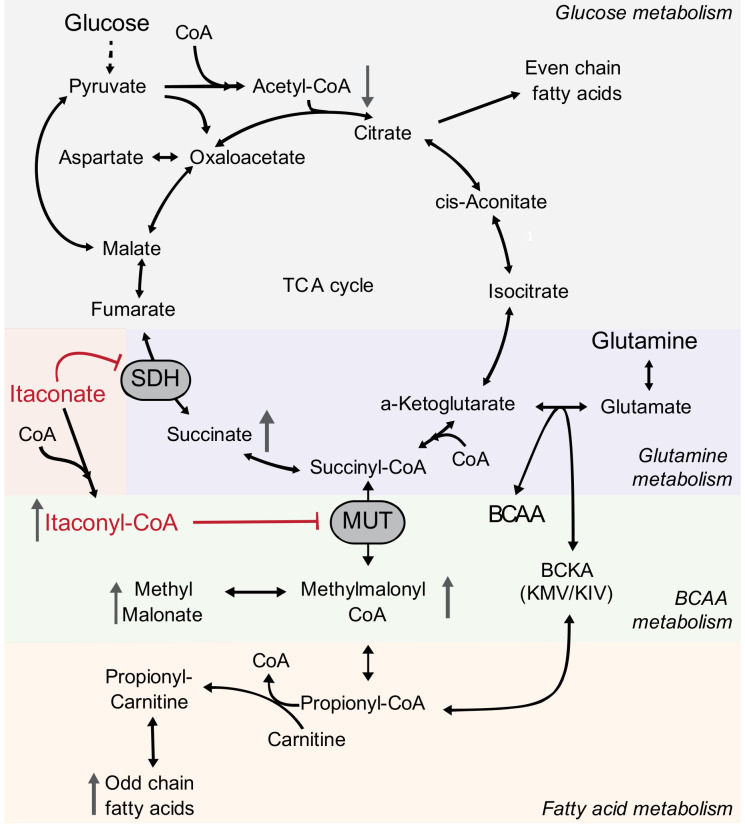
Metabolic compensation induced by itaconate metabolism. Schematic depicts the impact of itaconate metabolism on glucose, glutamine, branched-chain amino acid (BCAA), CoA species, and fatty acid metabolism in mammalian cells.

The fatty acid data above further suggest that itaconate influences CoA metabolism, as OCFA synthesis is driven by alterations in the availability of acetyl-CoA and propionyl-CoA for biosynthesis. To more directly characterize how itaconate influences the balance of CoAs in cells, we next quantified various species in Huh7 cells exposed to extracellular itaconate. We observed that itaconate decreased acetyl-CoA levels while propionyl-CoA and CoA levels were not affected (Figure 5d). Itaconate, therefore, increased the propionyl-CoA to acetyl-CoA ratio (Figure 5e). Importantly, carnitine acetyltransferase (CrAT) facilitates the transfer of CoAs out of the mitochondrial matrix to drive OCFA and mmBCFA synthesis [34], and the ratio of propionyl-carnitine to acetyl-carnitine was also significantly increased upon itaconate treatment (Figure 5f and Figure S5d). Collectively, our data suggest that itaconate drives the accumulation of alternate CoA species such as itaconyl-CoA that subsequently alters the relative abundance of compartment-specific CoA pools (i.e., cytosolic propionyl-CoA/acetyl-CoA). Through its impacts on SDH and MUT, and beyond, itaconate drives changes in the balance of CoA species and fatty acid diversity (Figure 6). 

## 3. Discussion

Our metabolic studies demonstrated that itaconate is a metabolic regulator of SDH and MUT activity, and targets reverberate through the metabolic network to impact TCA, amino acid, vitamin B_12_, CoA/carnitine, and fatty acid metabolism. Similar metabolic alterations were observed in cell types from diverse tissues, suggesting that therapeutic applications of itaconate could have numerous impacts in the body. By mapping the metabolic changes associated with itaconate treatment across these pathways, our study may provide useful information for understanding the role of both endogenously synthesized itaconate and exogenous itaconate treatments.

We observed that itaconate is taken up by diverse cell types inducing succinate accumulation. Using real-time metabolic flux analysis, we discovered that itaconate competed with succinate as substrates for SDH activity that was rapidly reversible (Figure 2). Dynamic (i.e., reversible) regulation of SDH activity may facilitate more precise execution and control of the inflammatory response, consisting of a two-stage metabolic remodeling [36]. Since our data depict that SDH inhibition is reversible and occurred within seconds, SDH might be an early target of itaconate to influence metabolism and cell function rapidly. This metabolic flexibility may be beneficial in numerous disease settings where transient SDH inhibition could modulate the inflammatory response [37] and mitigate tissue damage associated with reperfusion injury [22]. Succinate is an important signaling molecule [38,39] and the dynamic control of succinate concentration may further influence signaling events [40,41] and immune cell function [42,43].

Our metabolic studies indicate a regulatory impact of itaconate on propionate metabolism via inhibition of MUT flux induced by itaconyl-CoA (thereby increasing methylmalonate OCFA levels). MUT activity is coenzyme B_12_-dependent and, thus, the level of methylmalonate is an indirect measurement of coenzyme B_12_ levels. MUT catalyzes the reaction from methylmalonyl-CoA into the TCA cycle intermediate succinyl-CoA. Decreased MUT activity limited carbon flux into the TCA cycle and substrate availability for the SDH reaction. Further, propionyl-CoA is an important precursor for OCFAs [32,33,34]. To metabolize excess propionyl-CoA, free carnitine is esterified to generate propionyl-carnitine that is transported into the cytosol for fatty acid synthesis [44]. Thus, itaconate-exposed cells could exhibit imbalances in CoA and carnitine pools to influence other pathways.

Though itaconate degradation has been described in bacteria [17,45] and mammals [12,29], we did not observe itaconate degradation into pyruvate or TCA cycle intermediates in our cell models. However, we observed the accumulation of itaconyl-CoA. Itaconate activation to itaconyl-CoA may function as a “CoA sink” that impacts other CoA-dependent processes. Notably, etomoxir influences CoA homeostasis that further altered macrophage polarization and immune response [46]. In addition, itaconate activation to itaconyl-CoA might be catalyzed by succinyl-CoA synthetase (SCS) [29] activity and, thus, itaconate degradation may deplete mitochondrial substrate-level phosphorylation reaction [11]. More research is needed to distinguish the metabolic impact of itaconate and other potential degradation products, including itaconyl-CoA, citramalate, and mesaconate [12,29,47]. As such, itaconate degradation pathway is an exciting future research direction to discover novel metabolites with potential bioactive and regulatory functions.

An anti-inflammatory role for synthetic, cell-permeable derivatives of itaconate has been described by regulating Nrf2 and ATF3 depending pathways [14,15]. However, the impacts of natural itaconate on inflammation and succinate accumulation are different [48,49], indicating that itaconate metabolism may influence distinct metabolic and biological pathways. Specifically, we observed that the synthetic itaconate-derivative DMI did not alter SDH or MUT activity and fatty acid metabolism in our cell systems. As such, itaconate-induced metabolic reprograming may be a key driver facilitating the execution of inflammatory responses that is distinct from synthetic itaconate derivatives.

Collectively, our data indicate that itaconate metabolism influences diverse metabolic pathways, via SDH and MUT inhibition including central carbon, vitamin, amino acid, and fatty acid metabolism. Elucidating the function of metabolites involved in itaconate metabolism is an essential step to understanding the biological importance of itaconate in human health and disease. Ultimately, treatment strategies with itaconate may influence specific metabolic pathways at the systems level, and broader tools for studying metabolism may prove useful in studying this metabolite [50,51]. 

## 4. Materials and Methods 

### 4.1. Materials and Reagents

Media and sera were purchased from Life Technologies (Carlsbad, CA, USA) unless otherwise stated. Glucose and amino acid isotope tracers were purchased from Cambridge isotopes Inc. All other reagents were purchased from Sigma Aldrich (St. Louis, MO, USA) unless otherwise stated. 

### 4.2. Cell Culture

The following cell lines were used for the experiments: RAW264.7 (ATCC TIB-71), A549 (ATCC CCL-185), MCF7 (ATCC HTB-22), HepG2 (ATCC HB-8065), and Huh7 (provided by M. Hermann, MIT, Cambridge, MA, USA). All cell lines were tested negative for Mycoplasma contamination. Cells were cultured in DMEM medium containing 25 mM glucose, 10% FBS, 100 U/mL penicillin, and 100 µg/mL streptomycin in a humidified cell culture incubator at 37 °C and 5% CO_2_. Primary cortical neurons and astrocytes were isolated and cultured in Neurobasal medium (neurons) or DMEM medium containing 10%FBS, 10 mM glucose and 0 mM glutamine (astrocytes) as described in detail by Cordes et al. [22]. All media were adjusted to pH = 7.3.

### 4.3. Small Molecule Treatments

Cells were cultured in 12-well culture plates and exposed to itaconate (Sigma-Aldrich, St. Louis, MO, USA, Cat.#I29204) or dimethyl-itaconate (DMI, Sigma-Aldrich, St. Louis, MO, USA, Cat.#592498) as indicated in each figure legend. RAW264.7 cells were activated with 10 ng/mL lipopolysaccharide (LPS) for 24 h as indicated in the figure legend. Cells were cultured in a growth medium containing 500 nM cobalamin (Vitamin B_12_, Sigma-Aldrich, St. Louis, MO, USA, Cat.#V2876) when noted.

### 4.4. Cell Viability and Growth Assays

Cells were cultured in 96 well plates in the presence of increasing itaconate or DMI concentrations as indicated in the figure. Cell viability was determined using PrestoBlue Cell viability Reagent (Invitrogen, Carlsbad, CA, USA) per manufacturer instructions. 

### 4.5. Isotopic Tracing

Cells were cultured in medium containing stable isotope tracers of choice as indicated in the text. Tracers were purchased from Cambridge Isotopes Inc. (Tewksbury, MA, USA): [U-^13^C_6_]glucose (CLM-1396-25), [U-^13^C_5_]glutamine (CLM-1822-H-PK), [U-^13^C_6_]isoleucine (CLM-2248-H-0.1), [α-^15^N]glutamine (NLM-1016-1). For glucose and glutamine isotopic labeling experiments, cells were cultured in tracer medium as indicated in each figure legend. For glucose and glutamine tracing studies, cells were cultured in DMEM (Sigma-Aldrich, St. Louis, MO, USA, Cat.#5030) medium. ^12^C glucose was replaced with 25 mM [U-^13^C_6_]glucose and ^12^C glutamine was replaced with 4 mM [U-^13^C_5_] or [α-^15^N]glutamine, respectively. 

For tracing with labeled isoleucine, custom DMEM (HyClone Laboratories Inc, Logan, UT, USA) was formulated with 0.8 mM [U-^13^C_6_]isoleucine tracer and unlabeled versions of other chemical components. For ^13^C itaconate studies, cells were cultured in growth medium supplemented with 2 mM [U-^13^C_5_]itaconate (tracer was provided by National Institutes of Health (NIH) Metabolite Standards Synthesis Core) as indicated in the text. All media were supplemented with 10% FBS, 100 U/mL penicillin, and 100 µg/mL streptomycin and adjusted to pH = 7.3. Metabolites were extracted at the indicated time periods as indicated in the figure legends.

Mass isotopomer distributions and total metabolite abundances were computed by integrating mass fragments using a MATLAB based algorithm with corrections for natural isotope abundances as described previously [52,53]. Labeling is depicted as isotopologue distributions or as the labeled fraction of metabolites (mole percent enrichment (MPE) as previously described [53]. The contribution of ^13^C substrate to lipogenic Acetyl-CoA pool and fraction of newly synthesized palmitate was determined via isotopomer spectral analysis (ISA) using INCA as previously described [35,53]. Significance was considered as non-overlapping confidence intervals. Details on specific fragments are provided elsewhere [53].

### 4.6. Gas Chromatograph-Mass Spectrometry (GC-MS), Sample Preparation, and Analysis

Metabolites were extracted, analyzed, and quantified, as previously described in detail [53]. Briefly, cells were washed with saline solution and quenched with 0.25 mL −20 °C methanol. After adding 0.1 mL 4 °C cold water, cells were collected in tubes containing 0.25 mL −20 °C chloroform. The extracts were vortexed for 10 min at 4 °C and centrifuged at 16,000× *g* for 5 min at 4 °C. The upper aqueous phase was evaporated under vacuum at 4 °C. Derivatization for polar metabolites was performed using a Gerstel MPS with 15 μL of 2% (*w*/*v*) methoxyamine hydrochloride (Thermo Scientific) in pyridine (incubated for 60 min at 45 °C) and 15 μL N-tertbutyldimethylsilyl-N-methyltrifluoroacetamide (MTBSTFA) with 1% tert-butyldimethylchlorosilane (Regis Technologies, Morton Grove, IL, USA) (incubated further for 30 min at 45 °C). Derivatives were analyzed by GC-MS using a DB-35MS column (30 m × 0.25 i.d. × 0.25 μM) installed in an Agilent 7890B gas chromatograph (GC) interfaced with an Agilent 5977A mass spectrometer (MS) operating under electron impact ionization at 70 eV. The MS source was held at 230 °C and the quadrupole at 150 °C and helium was used as carrier gas. The GC oven was held at 100 °C for 2 min, increased to 300 °C at 10 °C/min and held for 4 min, and held at 325 °C for 3 min.

The lower organic phase was derivatized to form fatty acid methyl esters (FAMES) using 500 μL 2% H_2_SO_4_ in MeOH and incubation at 50 °C for 2 h. FAMES were extracted via addition of 100 μL saturated salt solution and 500 μL hexane. FAMES were analyzed using a Select FAME column (100 m × 0.25 mm i.d.) installed in an Agilent 7890 A GC interfaced with an Agilent 5975C MS. Helium was used as a carrier gas and the GC oven was held at 80 °C, increased by 20 °C/min to 170 °C, increased by 1 °C/min to 204 °C, then 20 °C/min to 250 °C and hold for 10 min.

### 4.7. Measurements Of CoA and Carnitine Species 

Coenzyme A and carnitine species were measured using reversed-phase liquid chromatography (RPLC) method, as previously described in detail [54]. Briefly, cells were washed with saline solution and quenched with 1 mL −20 °C 80% methanol/water, and the plate was transferred to the −80 °C freezer and incubated for 15 min. Cells were collected in tubes and the extracts were centrifuged at 16,000× *g* for 10 min at 4 °C. The extracts were dried under airflow, resuspended in 75 μL Buffer A and 5 µL of the sample was measured on a liquid chromatography (LC) coupled to a Q Exactive system (Q Exactive Hybrid Quadrupole-Orbitrab MS w/Vanquish Flex Binary UHPLC system, Thermo Scientific, Waltham, MA, USA). A C18 column (C18 1.7 µm, 100Å, 100 × 2.1 mm, Phenomenex, Cat.#00D-4475-AN) was employed with mobile phase Buffer A (5 mM ammonium acetate in water, pH = 6.8) and Buffer B (100% methanol). The QE-MS was operated in positive mode. Metabolites were verified with external standards or specific MS2 fragments (Table 1, Figure S6). Mass accuracy obtained for all metabolites was below 5 ppm. Data were acquired with Thermo Xcalibur software and analyzed using EL-Maven software [55]. Itaconyl-CoA was also identified based on specific labeling pattern derived from ^12^C and ^13^C itaconate and compared to non-treated cells. Itaconyl-CoA was detectable in itaconate-treated cells only and was M+5 labeled from [U-^13^C_5_]itaconate.

### 4.8. Respirometry

Respiration was measured in adherent monolayers of cells using a Seahorse XFe96 Analyzer with a minimum of 5 cellular replicates per plate. To measure succinate-driven respiration (Complex II, SDH), cells were permeabilized with 3 nM recombinant perfringolysin O (rPFO, commercially XF Plasma membrane permeabilizer (PMP), Agilent Technologies, Santa Clara, CA, USA) as previously described [9,22,56]. Permeabilized cells were offered 5 mM succinate as oxidizable substrate plus 4 mM ADP and initial oxygen consumption was measured, followed by injection of 0 or 5 mM itaconate (Port A), 2 μM oligomycin (Oligo) (Port B), 2 μM FCCP (Port C) and then addition of 0.5 μM rotenone and 1 μM antimycin (Ant/Rot) (Port D). For competition assays, 5 mM itaconate or 5 mM succinate were added via ports A-D as indicated in each figure. All assays were performed with 2 μM rotenone present in the assay medium and all media were adjusted to pH = 7.3 using KOH.

### 4.9. Statistics

Statistical analysis was performed using GraphPad Prism 7. The type and number of replicates and the statistical test used are described in each figure legends. Data are presented as means ± s.e.m. or box (25th to 75th percentile with median line) and whiskers (min. to max. values) as described in figure legends. Data obtained from ISA model are depicted as 95% confidence intervals and significance was considered as non-overlapping confidence intervals. Tissue culture was conducted in 12-well tissue culture plates, with 3 cellular replicates. Seahorse assays were performed in 96-well plates, with a minimum of 5 cellular wells per condition. All data are depicted from one representative experiment and each experiment was independently repeated two or more times. *p* values were calculated using a Student’s two-tailed *t*-test, one-way ANOVA or two-way ANOVA with no adjustment for multiple comparisons and * *p* value < 0.05; ** *p* < 0.01; *** *p* value < 0.001 and # *p* value < 0.0001.

## Figures and Tables

**Table 1:** Identification of CoA and carnitine species using Q Exactive system.

	Formula	Retention Time	Theoretical *m*/*z*	*m*/*z* H^+^	*m*/*z* detected (±5 ppm)	MS2 *m*/*z*
Coenzyme A	C_21_H_36_N_7_O_16_P_3_S	2.31 min	767.1152	768.1225	768.1216	261.127
Acetyl-CoA	C_23_H_38_N_7_O_17_P_3_S	5.44 min	809.1258	810.1331	810.1317	303.137
Propionyl-CoA	C_24_H_40_N_7_O_17_P_3_S	5.89 min	823.1414	824.1487	824.1477	317.153
Succinyl-CoA/Methylmalonyl-CoA	C_25_H_40_N_7_O_19_P_3_S	2.11 min	867.1313	880.1386	868.1379	317.154
Itaconyl-CoA	C_26_H_40_N_7_O_19_P_3_S	2.65 min	879.1313	880.1386	880.1379	N/A
Acetyl-Carnitine	C_9_H_17_NO_4_	1.23 min	203.1158	204.123	204.1232	85.028
Propionyl-Carnitine	C_10_H_19_NO_4_	1.64 min	217.1314	218.1387	218.1385	85.028

## Data Availability

The data presented in this study are available on request from the corresponding author.

## Supplementary Materials

The following are available online at https://www.mdpi.com/2218-1989/11/2/117/s1, Figure S1: Itaconate alters succinate levels in diverse cell types, Figure S2: Itaconate is a reversible SDH inhibitor, Figure S3: Itaconate modulates glutamine and glucose metabolism, Figure S4: Itaconate influences methylmalonyl-CoA mutase activity, Figure S5: Itaconate alters fatty acid metabolism, Figure S6: LC-MS spectra of CoA and carnitine species.
