# Supplementary material for: Itaconate Alters Succinate and Coenzyme A Metabolism via Inhibition of Mitochondrial Complex II and Methylmalonyl-CoA Mutase
Source: Metabolites. 2021 Feb 18;11(2):117. doi: 10.3390/metabo11020117 (PMC7922098; doi:10.3390/metabo11020117)
Supplement: Supplementary file 1 [file metabolites-11-00117-s001.pdf]

metabolites MDPI

Supplementary Figures

Thekla Cordes[1] and Christian M. Metallo[1*]

[1] Department of Bioengineering, University of California, San Diego, 9500 Gilman Drive, 92093 La Jolla, CA, USA
* Correspondence: Christian M. Metallo (cmetallo@ucsd.edu)

Figure S1: Itaconate alters succinate levels in diverse cell types
Figure S2: Itaconate is a reversible SDH inhibitor
Figure S3: Itaconate modulates glutamine and glucose metabolism
Figure S4: Itaconate influences methylmalonyl-CoA mutase activity
Figure S5: Itaconate alters fatty acid metabolism
Figure S6: LC-MS mass spectra of CoA and carnitine species

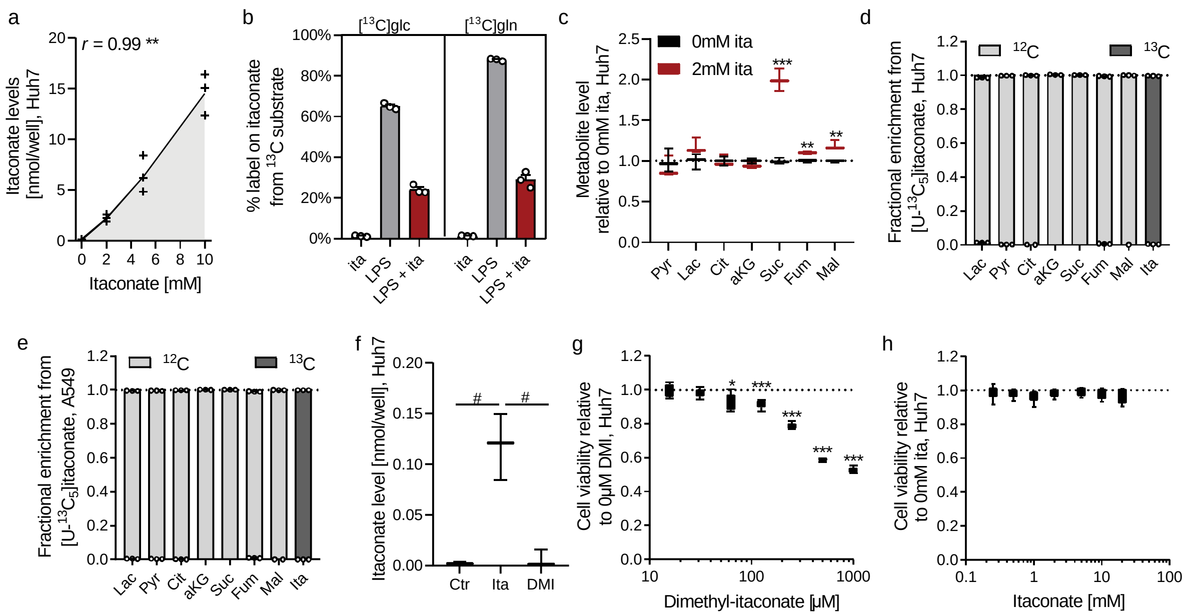

Figure S1. Itaconate promotes succinate accumulation in diverse cell types.

(a) Intracellular itaconate levels in Huh7 cells exposed to increasing exogenous itaconate for 48h. Pearson correlation coefficient (*r*) of itaconate levels.

(b) Labeling on itaconate from[U-$^{13}C_5$]glutamine and [U-$^{13}C_6$]glucose in RAW264.7 cells exposed to LPS or 2 mM extracellular itaconate for 24h.

(c) Itaconate increased succinate levels in Huh7 cells exposed to 2mM itaconate for 48h.

(d) Labeling on metabolites in Huh7 cultured in the presence of 2mM [U-$^{13}C_5$]itaconate for 48h.

(e) Labeling on metabolites in A549 cultured in the presence of 2mM [U-$^{13}C_5$]itaconate for 48h.

(f) Itaconate levels in Huh7 cells exposed to 2mM itaconate or 62.5μM DMI for 48h.

(g) Cell viability (PrestoBlue assay) in Huh7 cells exposed to increasing dimethyl-itaconate (DMI) levels for 48h, Significance relative to 0mM DMI.

(h) Cell viability (PrestoBlue assay) in Huh7 cells exposed to increasing itaconate concentrations for 48h

Data are depicted as mean ± s.e.m. (**a**, **b**, **d**, **e**) or box and whiskers (**c**, **f**, **g**, **h**) obtained from 3 (**a**-**f**) or 6 (**g**, **h**) cellular replicates. Students t-test (**c**) or one-way ANOVA (**f**) with no adjustment for multiple comparisons and * $P < 0.05$, ** $P < 0.01$, *** $P < 0.001$, # $P < 0.0001$.

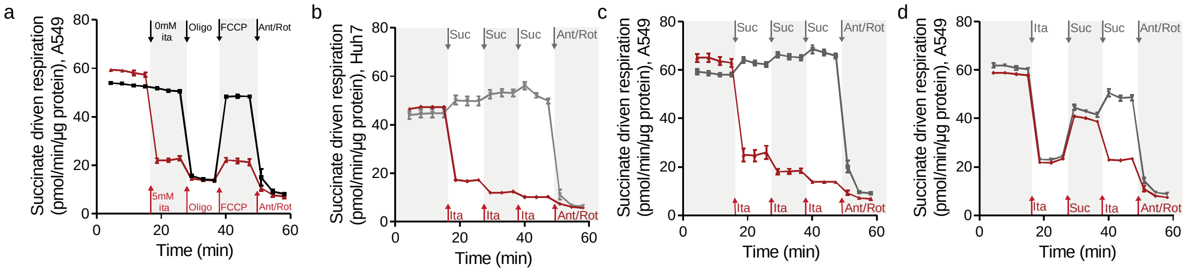

Figure S2. Itaconate is a reversible SDH inhibitor.

(a) Succinate driven respiration in A549 cells in the presence (red) or absence (black) of itaconate.

(b) Succinate driven respiration with increasing itaconate (red) or succinate (grey) concentrations in Huh7 cells.

(c) Succinate driven respiration with increasing itaconate (red) or succinate (grey) concentrations in A549 cells.

(d) Succinate driven respiration after serial addition of Ita/Suc/Ita (red) or Ita/Suc/Suc (grey) in A549 cells.

Data are depicted as mean ± s.e.m. obtained from 5 cellular replicates.

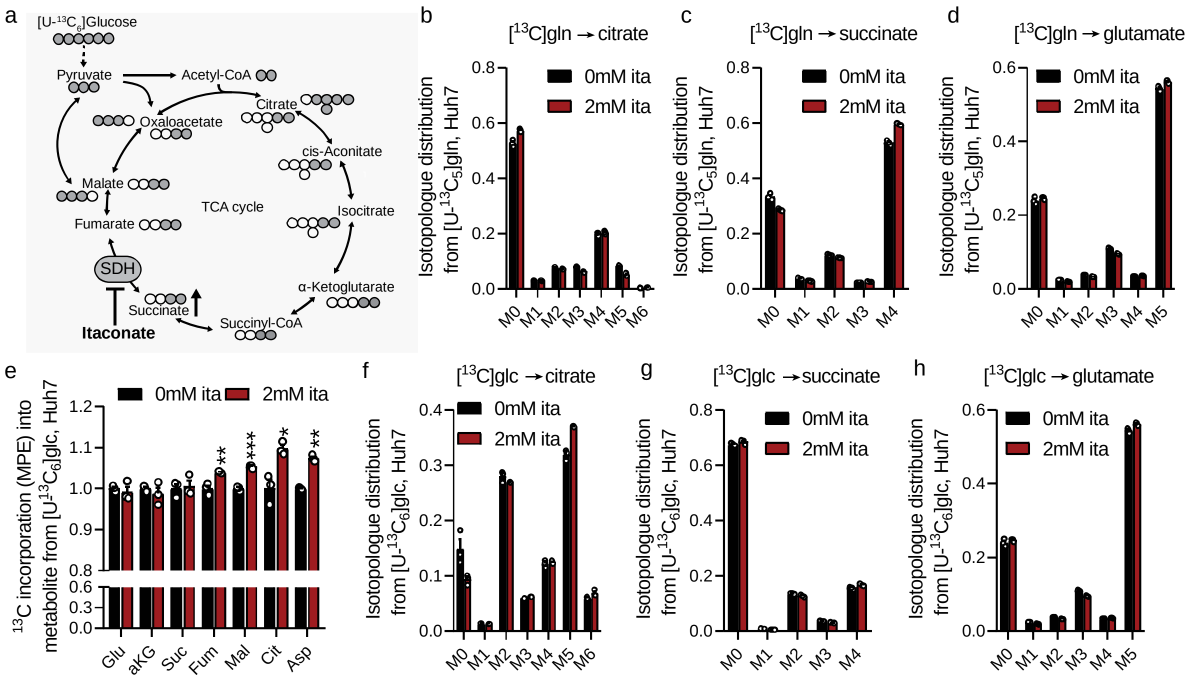

Figure S3. Itaconate modulates glutamine and glucose metabolism

(a) Schematic depicting carbon incorporation into TCA cycle intermediates from [U-$^{13}C_6$]glucose. Open circles depict ^{12}C, closed circles ^{13}C carbons.

(b) Isotopologue distribution on citrate from [U-$^{13}C_5$]glutamine in Huh7 cells cultured for 48h.

(c) Isotopologue distribution on succinate from [U-$^{13}C_5$]glutamine in Huh7 cells cultured for 48h.

(d) Isotopologue distribution on glutamate from [U-$^{13}C_5$]glutamine in Huh7 cells cultured for 48h.

(e) ^{13}C incorporation (mole percent enrichment) into metabolites from [U-$^{13}C_6$]glucose in Huh7 cells cultured for 48h.

(f) Isotopologue distribution on citrate from [U-$^{13}C_6$]glucose in Huh7 cells cultured for 48h.

(g) Isotopologue distribution on succinate from [U-$^{13}C_6$]glucose in Huh7 cells cultured for 48h.

(h) Isotopologue distribution on glutamate from [U-$^{13}C_6$]glucose in Huh7 cells cultured for 48h.

Data are depicted as mean ± s.e.m. obtained from 3 cellular replicates. Students t-test (**e**) with * $P < 0.05$, ** $P < 0.01$, *** $P < 0.001$

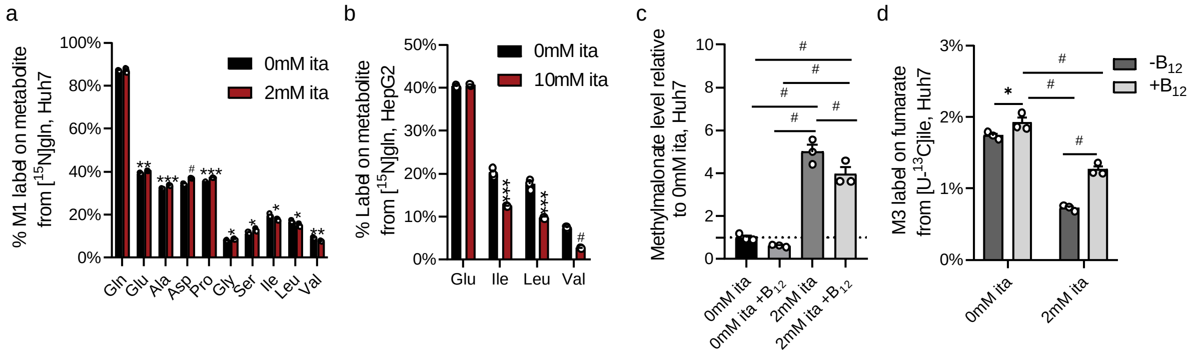

Figure S4. Itaconate influences methylmalonyl-CoA mutase (MUT) activity.

(a) Labeling on amino acids from [α-^{15}N]glutamine in Huh7 cells cultured for 48h.

(b) Labeling on amino acids from [α-^{15}N]glutamine in HepG2 cells cultured for 48h.

(c) Methylmalonate level in Huh7 cells cultured with 500 nM Vitamin B_{12} addition in the presence of 2 mM itaconate for 48h.

(d) M3 label on fumarate from [U-$^{13}C_6$]isoleucine in Huh7 and HepG2 cells exposed to 2 mM itaconate for 48h.

Data are depicted as mean ± s.e.m. obtained from 3 cellular replicates. Students *t*-test (**a**, **b**) or one-way ANOVA (**c**, **d**) with no adjustment for multiple comparisons and * $P < 0.05$, ** $P < 0.01$, *** $P < 0.001$, # $P < 0.0001$.

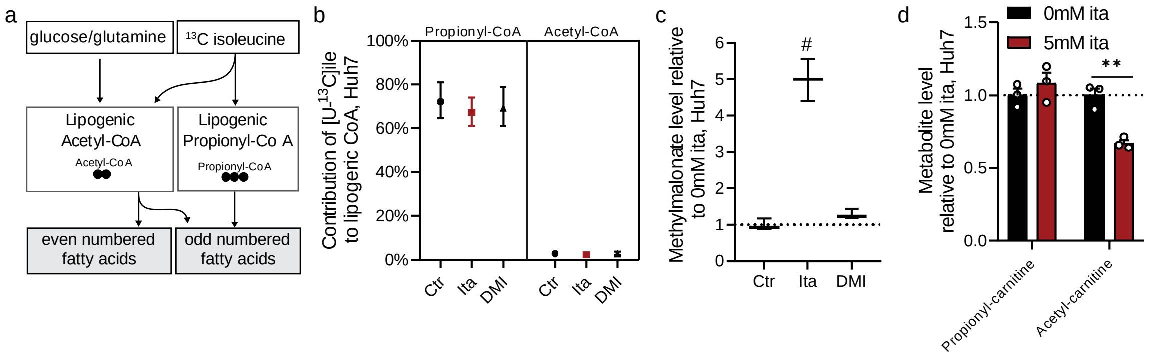

Figure S5. Itaconate alters fatty acid metabolism.
(a) Schematic depicting carbon usage from ^{13}C glucose and ^{13}C isoleucine for *de novo* lipogenesis.
(b) Contribution of [U-$^{13}C_6$]isoleucine to lipogenic propionyl and acetyl-CoA pool in Huh7 cells at 48h.
(c) Methylmalonate levels in Huh7 cells exposed to 2 mM itaconate or 62.5 μM dimethyl-itaconate (DMI) for 48h.
(d) Metabolite abundance of acetyl-carnitine and propionyl-carnitine in Huh7 cells cultured for 48h.

Data are depicted as 95% confidence intervals from ISA model (**b**), box and whiskers (**c**) or mean ± s.e.m. (**d**) obtained from 3 cellular replicates. Students *t*-test (**d**) or one-way ANOVA (**c**) with * $P < 0.05$, ** $P < 0.01$, *** $P < 0.001$, # $P < 0.0001$. Significance was considered as non-overlapping confidence intervals for **b.**

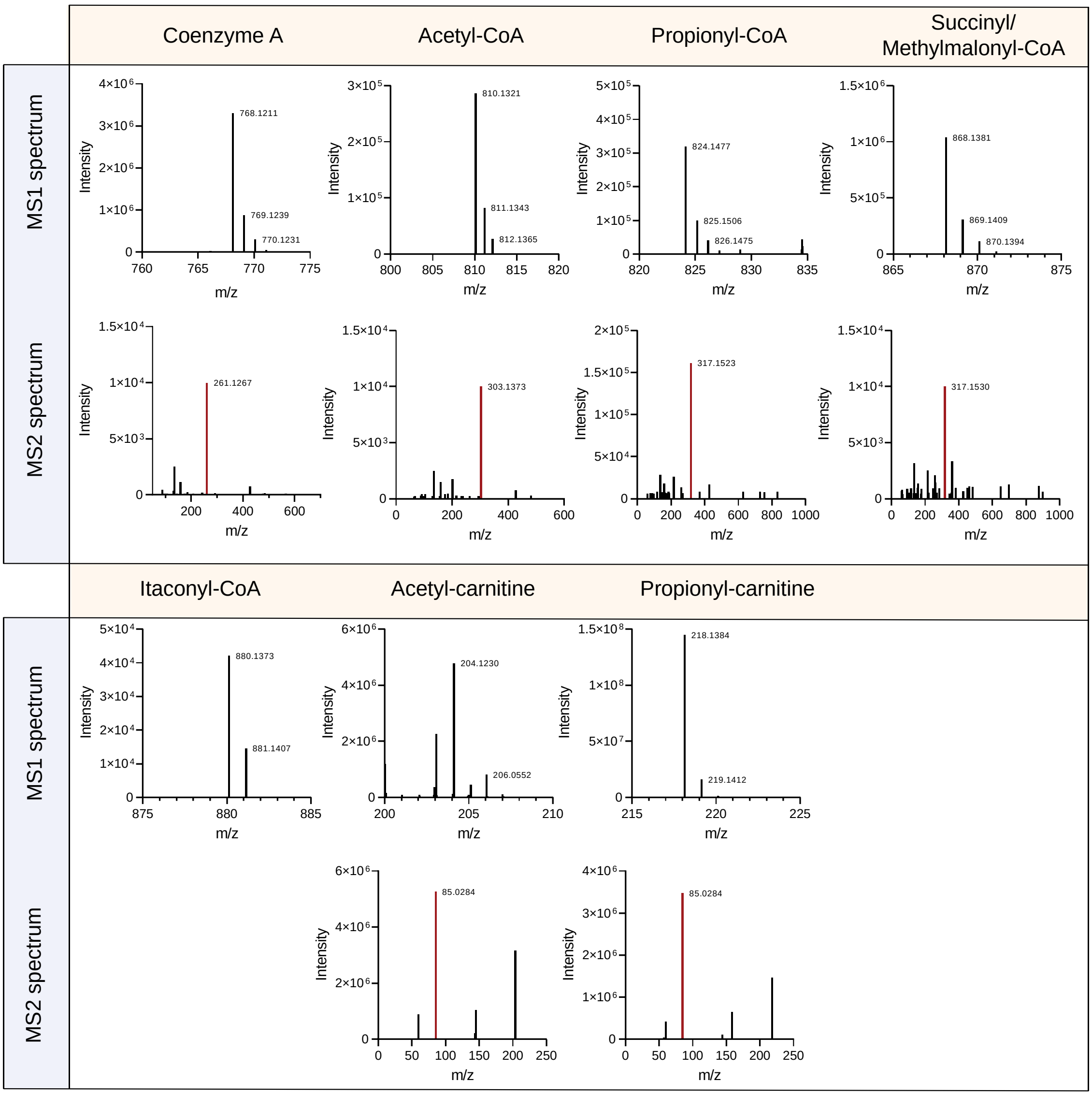

Figure S6. LC-MS mass spectra of CoA and carnitine species.
The figure depicts MS1 and MS2 spectra for each metabolite measure with LC - Q Exactive system.

© 2020 by the authors. Submitted for possible open access publication under the terms and conditions of the Creative Commons Attribution (CC BY) license (http://creativecommons.org/licenses/by/4.0/).
